# Morbidity and Mortality in 7,684 Women According to Personal Hair Dye Use: The Copenhagen City Heart Study followed for 37 Years

**DOI:** 10.1371/journal.pone.0151636

**Published:** 2016-03-17

**Authors:** Signe Vedel-Krogh, Sune F. Nielsen, Peter Schnohr, Børge G. Nordestgaard

**Affiliations:** 1 Department of Clinical Biochemistry, Herlev and Gentofte Hospitals, Copenhagen University Hospital, Copenhagen, Denmark; 2 Faculty of Health and Medical Sciences, University of Copenhagen, Copenhagen, Denmark; 3 The Copenhagen City Heart Study, Frederiksberg Hospital, Copenhagen University Hospital, Frederiksberg, Denmark; Federico II University, Naples, ITALY

## Abstract

**Background:**

Permanent hair dye contains aromatic amines which are carcinogenic, and can cause allergic skin reactions. In the long term personal use of hair dye might therefore influence both morbidity and mortality.

**Objectives:**

We tested the hypothesis that personal use of hair dye in women is associated with increased morbidity and mortality in the general population.

**Methods:**

We included 7,684 women from the Copenhagen City Heart Study with information on the use of personal hair dye. We assessed the risk of cancer, skin diseases, other morbidities, and mortality during a median follow-up of 27 years (range 0–37).

**Results:**

The multivariable adjusted hazard ratio for malignant melanoma in women with versus without personal use of hair dye was 2.07 (95% confidence interval 1.25–3.42). There was no increased risk of other cancer types. For other skin diseases and other major causes of morbidity we found no differences between the two groups, except for a minor excess of digestive diseases and increased risk of Parkinson’s disease among women using hair dye. Finally, we found no difference in all-cause mortality comparing women using personal hair dye or not. After correction for multiple comparisons, none of the results remained significant. However, in sensitivity analysis the excess risk of malignant melanoma remained increased with a hazard ratio of 2.58 (95%CI 1.33–5.03) among users of personal hair dye.

**Conclusions:**

Personal use of hair dye does not have major influences on morbidity and mortality. Our finding of a 2-fold risk of malignant melanoma in women using hair dye is hypothesis generating.

## Introduction

It is estimated that 50–80% of all women in the EU, the US, and in Japan have used hair dye [1]. In the 1970s, some aromatic amines contained in hair dye were banned from the European market due to studies of their carcinogenicity in animal models. Nonetheless, the latest report from the International Agency for Research on Cancer (IARC) concluded that epidemiological evidence regarding cancer risk following personal use of hair dye is inadequate, and therefore personal use of hair dye is presently not classifiable as carcinogenic in humans. Occupational exposures as a hairdresser or barber is, however, classified as probably carcinogenic to humans.

Besides their potential carcinogenicity, aromatic amines also have allergic potential [2], can cause asthma in hairdressers [3], and some are inducers of both pro- and anti-inflammatory immune responses in mice [4]. It is therefore plausible that personal use of hair dye could influence both morbidity and mortality in the long term, but no study has assessed such risk in the general population with the length of follow-up that might be necessary to detect risk.

Modern day hair dyes can be categorized as oxidative (permanent), direct (temporary or semi-permanent), metal salts, and naturel dyes [5]. According to the National Cancer Institute more than 5,000 different chemicals are used in hair dyes [6]. Oxidative hair dyes form up to 80% of the market in the EU and consist of three components: the primary intermediates (aromatic amines) that in the presence of an oxidizing agent react with the couplers to form a specific color [5]. The first patent of an oxidative hair dye was applied for in 1883 with the use of para-phenylenediamine or 2,5-diaminotoluene in combination with an oxidizing agent [7] based on the discovery that the colorless para-phenylenediamine produced a colored compound after oxidation. From the 1930s up until the 1970 several aromatic amines were used in oxidative hair dyes but during the 1970s there was a growing concern that some of these were biologically active. A study by Ames et al. published in 1975 found mutagenic activity in 10 out of a total of 18 amine components of oxidative hair dyes. The reported mutagenic components were 2,4-diaminoanisole, 2,4-diaminoanisone, 4-nitro-*o* -phenylenediamine, 2-nitro-*p*-phenylenediamine, 2-amino-5-nitrophenol, *m*-phenylenediamine, *o*-phenylenediamine, 2-amino-4-nitrophenol, 2,4-diaminotoluene, and 2,5-diaminotoluene [8]. Some of these components were also found to be mutagenic in rats by the U.S. National Cancer Institute [9]. In 1976 the first EU cosmetics directive was published [10] with a deadline for transposition in the member states by 1978 although extensions were granted for specified chemical compounds in the first amendment. This led to modification and removal of some ingredients of oxidative hair dyes from the European market. The directive also laid down concentration restrictions on other chemical compounds in hair dyes although carcinogenic compounds like 4-aminobiphenyl was banned from sale to the general public as late as in 1989 [1] and from cosmetics in 2004 [10]. The EU cosmetics directive has been amended several times since 1976. Today, the most widely used primary intermediates are phenylenediamine (PPD) and 2,5-toluenediamine (PTD) and they are permitted in the EU for use in hair coloring products as primary intermediates below a certain concentration and with mandatory warnings [11].

We tested the hypothesis that personal use of hair dye in women is associated with increased morbidity and mortality in the general population. For this purpose, we studied 7,864 apparently healthy women from the general population invited to participate in the Copenhagen City Heart Study and followed them for up to 37 years. During follow-up, 71% of the women died and 93% developed morbidity captured as a discharge diagnosis from hospitals or ambulatory care.

## Materials and Methods

### Study population

The Copenhagen City Heart Study is a prospective study of the general population of Copenhagen, Denmark, initiated in 1976–1978 with follow-up examinations in 1981–1983, 1991–1994, 2001–2003, and 2011–2014 [12,13]. Participants aged 20 years and older were randomly selected from the general population using the Danish civil registration system [14]. In the present study, we included participants from the 1976–78 examination. No participants were lost to follow-up. All participants completed a questionnaire, underwent a physical examination including spirometry, and provided blood samples. Participants reported on smoking habits, alcohol consumption, leisure-time physical activity, marital status, level of education, and income. Personal use of hair dye was determined as an affirmative response to the question “Do you dye your hair?”. Because only 15 of the 6,489 men with information on the use of hair dye at the 1976–1978 examination did use hair dye, we restricted our analyses to women only. We included 7,684 participants, 5,961 participants did not use hair dye, and 1,723 participants did use hair dye. All participants gave written informed consent.

The study was approved by the Danish ethics committee (KF V.100.2039/91, Copenhagen and Frederiksberg committee), and conducted according to the Declaration of Helsinki. All participants gave written informed consent.

### Morbidity and mortality

Based on the World Health Organization International Classification of Diseases 8^th^ and 10^th^ edition we prospectively analysed morbidity using the national Danish Patient Registry [15], which recorded all discharge diagnoses from Danish hospitals from 1977 including outpatients and emergency visits from 1995. Information on date of death was drawn from the national Danish Civil Registration system, which is 100% complete from 1968. Information on cancer was drawn from the national Danish Cancer Registry [16], which contains data on the incidence of cancer in the Danish population from 1943. (See S1 and S2 Tables for all diagnoses).

We grouped major morbidities based on the World Health Organization and the global burden of diseases [17]. As the use of hair dye has been postulated as a potential risk factor for systemic lupus erythematosus and rheumatoid arthritis [18,19], we chose to include these diseases as well as other autoimmune diseases such as Sjogren's syndrome, systemic sclerosis, polydermatomyositis in our analyses. We also included miscarriages, as an increased risk of miscarriages when working as a hairdresser has been reported [20]. Diagnoses of cancer were grouped as done previously [21]. In sub-analyses we looked at validated diagnoses of ischemic heart disease, ischemic cerebrovascular disease, spirometrically diagnosed chronic obstructive pulmonary disease, and self-reported asthma, as done previously [22–25]. We included Parkinson’s disease and Alzheimer’s dementia as these are two of the most common neuropsychiatric conditions [26,27], as well as diagnoses hypothesized to be associated with personal use of hair dye.

Ischemic heart disease (World Health Organization International Classification of Diseases: ICD8 410–414; ICD10 I20-I25) was collected and verified by reviewing all hospital admissions and diagnoses entered in the national Danish Patient Registry as well as medical records from hospitals and general practitioners [22,28]. Myocardial infarction and characteristic symptoms of angina pectoris was classified as ischemic heart disease.

Information on diagnosis of ischemic cerebrovascular disease (World Health Organization International Classification of Diseases ICD8 432–435; ICD10 I61-I69, G45) was collected by reviewing hospital admissions with diagnoses entered in the national Danish Patient Registry [23,29] and classified on the basis of sudden onset of focal neurological symptoms (ischemic stroke, transient ischemic attack or amaurosis fugax). Hemorrhagic stroke and subarachnoidal hemorrhage were excluded from ischemic cerebrovascular disease.

We identified chronic obstructive pulmonary disease spirometically at baseline [24]. We defined chronic obstructive pulmonary disease by the ratio of forced expiratory volume in 1 second (FEV_1_) and forced vital capacity (FVC) under the lower limit of normal (5^th^ percentile of a frequency distribution) excluding participants with self-reported asthma in accordance with recent recommendations [30,31]. Diagnosis of asthma at baseline was an affirmative answer to the question “Do you have asthma?”[25].

Hypersensitivity was as an affirmative answer to the question “Does food, medicine, grass, animals, flowers or the like give you asthma, rhinitis, itching, eczema, stomach problems or other symptoms?” [32]. We only had information regarding hypersensitivity from the 1981–83 examinations, so assessment of hypersensitivity was restricted to women who had answered the question regarding hypersensitivity at this examination (n = 5,933).

### Covariates

Several potential confounders were included in the analyses. Information on covariates was obtained at baseline. Participants were classified as never smokers, former smokers or current smokers. For former smokers and current smokers, pack-years of cigarettes smoked or equivalent were calculated. One pack-year corresponds to one pack of 20 cigarettes or equivalent smoked for one year. Information of systolic blood pressure, leisure-time physical activity per week, plasma triglycerides, and plasma cholesterol were included. Body mass index was calculated as measured weight (kg) divided by measured height (m) squared. Furthermore, we included alcohol consumption in units per week, plasma glucose, FEV_1_ in percent of the predicted value, and the ratio of FEV_1_/FVC. We included marital status, education, and income as socioeconomic markers. Information on age and sex was 100% complete, whereas information on other covariates were > 98% complete.

### Statistical analyses

Statistical analyses were done using STATA 13.1.

We had full information on age and birthday on all individuals. Imputation of missing covariates at baseline (<2%) was done by multivariable normal regression using an iterative Markov chain Monte Carl method and taking all available covariates into account; however, if only individuals with complete information on covariates were included, results were similar to those reported.

Follow-up for every participant began at study entry and ended at event, death (n = 5,464), emigration (n = 45), or end of follow-up, whichever came first. For all endpoints except cancer, follow-up ended 23^rd^ of April 2013. Cancer follow-up ended 1^st^ of January 2012, because the national Danish Cancer Registry lags slightly behind other registers in Denmark.

We did power calculations to determine the minimal detectable hazard ratio for both mortality and morbidity in a composite end-point. With a fixed sample size, that is, all women examined in 1976–1978, we used a two-sided Log-rank test, using the Freedman method, with a 0.05 alpha level and a power of 80% for both tests.

Cause-specific Cox proportional hazards regression models, with age as time scale, estimated hazard ratios with 95% confidence intervals for morbidity and mortality. Proportionality of hazards over time was assessed based on Schoenfeld residuals as well as visually by plotting –ln(–ln(survival)) versus ln(analysis time). No major violations of the proportional hazard assumption were noted. Logistic regression models estimated odds ratios with 95% confidence interval in cross-sectional analyses of chronic obstructive pulmonary disease, asthma and hypersensitivity. The models were multivariable adjusted for major risk factors for morbidity and mortality, that is for age (as timescale), birth year, body mass index, smoking, pack-years, alcohol consumption, leisure-time physical activity, marital status, level of education, level of income, systolic blood pressure, plasma cholesterol, plasma glucose, plasma triglycerides, FEV_1_ in percent of the predicted value, and FEV_1_/FVC. The analysis of diabetes mellitus was also multivariable adjusted, but plasma glucose at baseline was not added to the model. Predicted values for FEV_1_ were calculated for healthy, never smoking women using multiple linear regression analysis with age and height as covariates. The lower limit of normal for FEV_1_/FVC was defined as a ratio below the 5th percentile of the predicted ratio for height and age in healthy, never smoking women. When assessing the risk of chronic obstructive pulmonary disease the model was multivariable adjusted, but not for FEV_1_/FVC. Cumulative incidences were plotted using Kaplan-Meier curves and differences between women with or without use of hair dye was determined using Wald or log-rank test.

We tested for interaction of hair dye and covariates in the main models using the likelihood-ratio test.

In a sensitivity analysis, we calculated the sub-distribution hazard of malignant melanoma in a competing risk model using the method of Fine and Gray with death as the competing event.

## Results

### Study population

We included 7,684 women from the Copenhagen City Heart Study’s first examination in 1976–1978 and followed them until 23^rd^ of April 2013 (median 27 years, range 0 to 37). Cancer follow-up ended at 1^st^ of January 2012 (median 25 years, range 0 to 36). Women using personal hair dye had a slightly higher income than women not dying hair. This aside, there were no differences in baseline characteristics after Bonferroni correction for multiple comparisons (Table 1).

**Table 1:** Baseline characteristics of female participants according to personal use of hair dye.

	Not using hair dye	Using hair dye	P-value^a^
Number	5,961	1,723	
Age, years	54 (45–61)	53 (45–59)	0.004 ^NS^
Smoking			0.08
Never smoker	1,661 (28%)	454 (26%)	
Former smoker	883 (15%)	230 (13%)	
Current smoker	3,417 (57%)	1,039 (60%)	
Pack-years^b^	15 (8–24)	15 (8–23)	0.11
Systolic blood pressure, mmHg	132 (119–148)	131 (119–146)	0.26
Leisure-time physical activity per week			0.04 ^NS^
<2 hours	1,181 (20%)	345 (20%)	
2–4 hours light	3,505 (59%)	997 (58%)	
2–4 demanding	1,239 (21%)	359 (21%)	
>4 hours	36 (<1%)	22 (1%)	
Triglycerides, mg/dL	112 (83–159)	111 (84–153)	0.21
Cholesterol, mg/dL	239 (208–273)	237 (209–269)	0.17
Body Mass Index (kg/m^2^)			0.04 ^NS^
< 18.5	181 (3%)	40 (2%)	
18.5–24.9	3,364 (56%)	1,013 (59%)	
25.0–29.9	1,665 (28%)	489 (28%)	
>30	751 (13%)	181 (11%)	
Alcohol consumptions per week			0.24
<14 units	5,580 (94%)	1,599 (93%)	
≥14 units	381 (6%)	124 (7%)	
Glucose, mg/dL	110 (101–123)	110 (101–121)	0.03^NS^
FEV_1_% predicted	94 (82–107)	95 (83–107)	0.17
FEV_1_/FVC, %	81 (75–86)	81 (75–86)	0.81
Marital status			0.12
Single	1,184 (20%)	327 (19%)	
Married	3,486 (58%)	997 (59%)	
Separated or divorced	620 (11%)	201 (12%)	
Widow	671 (11%)	167 (10%)	
Level of education			0.61
Elementary (0–9 years)	4,319 (72%)	1,255 (73%)	
High school (10–12 years)	1,429 (24%)	415 (24%)	
Academic (> 12 years)	213 (4%)	53 (3%)	
Level of income per month			0.001
<4,000 Dkr	2,030 (33%)	512 (30%)	
4,000–10,000 Dkr	2,911 (49%)	873 (51%)	
>10,000 Dkr	1,020 (17%)	338 (19%)	

Data are n (%) or median (interquartile range). Baseline characteristics were at the date of examination in 1976–78.

^a^P-values are calculated using Wilcoxon rank-sum or Pearson's chi-squared test.

^b^ Pack-years are calculated for current and former smokers.

^NS^ = Not significant when controlled for 15 multiple comparisons using the Bonferroni method (required p-value = 0.05/15 = 0.003 = 3∙10^−3^). To convert cholesterol levels in mg/dL to mmol/L, multiply by 0.02586. To convert glucose levels in mg/dL to mmol/L, multiply by 0.0555. To convert triglycerides in mg/dL to mmol/L, multiply by 0.01129. FEV_1_ (forced expiratory volume in 1 second). FVC (forced vital capacity).

### Cancer

**Fig 1:**
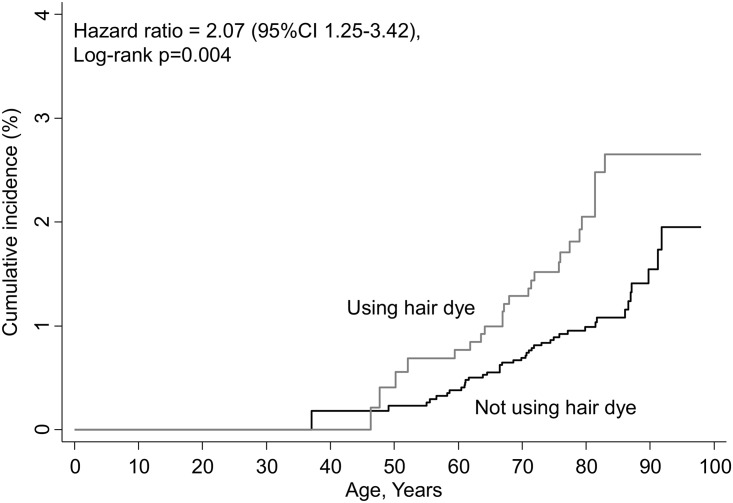
Cumulative incidence of malignant melanoma in women using hair dye compared to women not using hair dye at baseline, the 1976–1978 examination. Follow up began at the 1976–1978 examination and ended at event (n = 65), death (n = 5,200), emigration (n = 45) or 1^st^ of January 2012. Age is used as timescale.

The multivariable adjusted hazard ratio for malignant melanoma in women with versus without personal hair dye use was 2.07 (1.25–3.42), log-rank p-value 0.004 (Fig 1); however, the result did not remain significant after Bonferroni correction for 27 multiple comparisons (Table 2). During follow-up, 2,637 (34%) participants developed cancer. We found no increased risk of other types of cancer.

**Table 2:** Cancer according to personal use of hair dye during up to 36 years of follow-up in women from the general population.

	Not using hair dye, n = 5,961	Using hair dye, n = 1,723	Hazard ratio(95% confidence interval)	P-value^a^
	No. of events	No. of events		
Malignant melanoma	40	25	2.07 (1.25, 3.42)	0.005 ^NS^
Other skin cancer	410	141	1.10 (0.91, 1.33)	0.33
Non-skin cancer	1,669	515	1.00 (0.91, 1.11)	0.98
**Respiratory cancers**				
Lung cancer	269	84	1.02 (0.80, 1.31)	0.87
Pharynx cancer	31	8	0.95 (0.43, 2.08)	0.89
Larynx cancer	16	4	0.82 (0.27, 2.45)	0.72
**Gastrointestinal cancers**				
Colon cancer	257	90	1.15 (0.91, 1.47)	0.24
Pancreas cancer	77	28	1.20 (0.78, 1.86)	0.41
Liver cancer	37	13	1.18 (0.63, 2.24)	0.61
Stomach cancer	35	8	0.74 (0.34, 1.61)	0.45
Oesophagus cancer	13	4	0.86 (0.27, 2.67)	0.79
Small intestine cancer	8	1	0.37 (0.05, 3.00)	0.35
**Haematological cancers**				
Leukaemia	40	5	0.40 (0.16, 1.02)	0.06
Multiple myeloma	17	5	0.92 (0.34, 2.52)	0.87
Non-Hodgkin’s lymphoma	11	1	0.34 (0.04, 2.73)	0.31
Hodgkin’s lymphoma	1	0	-	-
**Women’s cancers**				
Breast cancer	461	151	1.05 (0.87, 1.26)	0.60
Ovary cancer	111	39	1.15 (0.80, 1.66)	0.46
Corpus uteri cancer	106	41	1.26 (0.88, 1.81)	0.21
Cervix uteri cancer	85	23	0.90 (0.57, 1.43)	0.66
**Other cancers**				
Urinary cancer	48	12	0.81 (0.43, 1.53)	0.52
Kidney cancer	29	5	0.55 (0.21, 1.42)	0.22
Brain cancer	31	10	1.02 (0.50, 2.09)	0.95
Sarcoma	15	4	0.80 (0.26, 2.44)	0.70
Endocrine cancers	12	1	0.29 (0.04, 2.23)	0.23
Remaining cancers	37	11	0.92 (0.47, 1.81)	0.81
Any cancer	2002	635	1.04 (0.95, 1.13)	0.43

Hazard ratios are multivariable adjusted for age, birth year, body mass index, smoking, pack-years, alcohol consumption, leisure-time physical activity, marital status, level of education, level of income, systolic blood pressure, plasma cholesterol, plasma triglycerides, plasma glucose, forced expiratory volume in one second (FEV_1_) percent of predicted value, and FEV_1_/Forced vital capacity (FVC). Any cancer is first diagnosis of cancer; however, individuals can have more than one cancer diagnosis and therefore the sum of the separate cancer types will be greater than the number of any cancer. Likewise, non-skin cancer is first diagnosed non-skin cancer and skin-cancer is the first diagnosed skin cancer.

^NS^ = Not significant when controlled for 27 multiple comparisons (required p value = 0.05/27 = 0.002).

^a^ = Wald’s test.

In our main models we did not find any significant tests for interaction after Bonferroni correction.

Using a competing risk model with death as the competing event, the sub-distribution hazard of malignant melanoma was 2.02 (CI 1.22–3.26) in women using personal hair dye compared with women not using hair dye.

**Fig 2:**
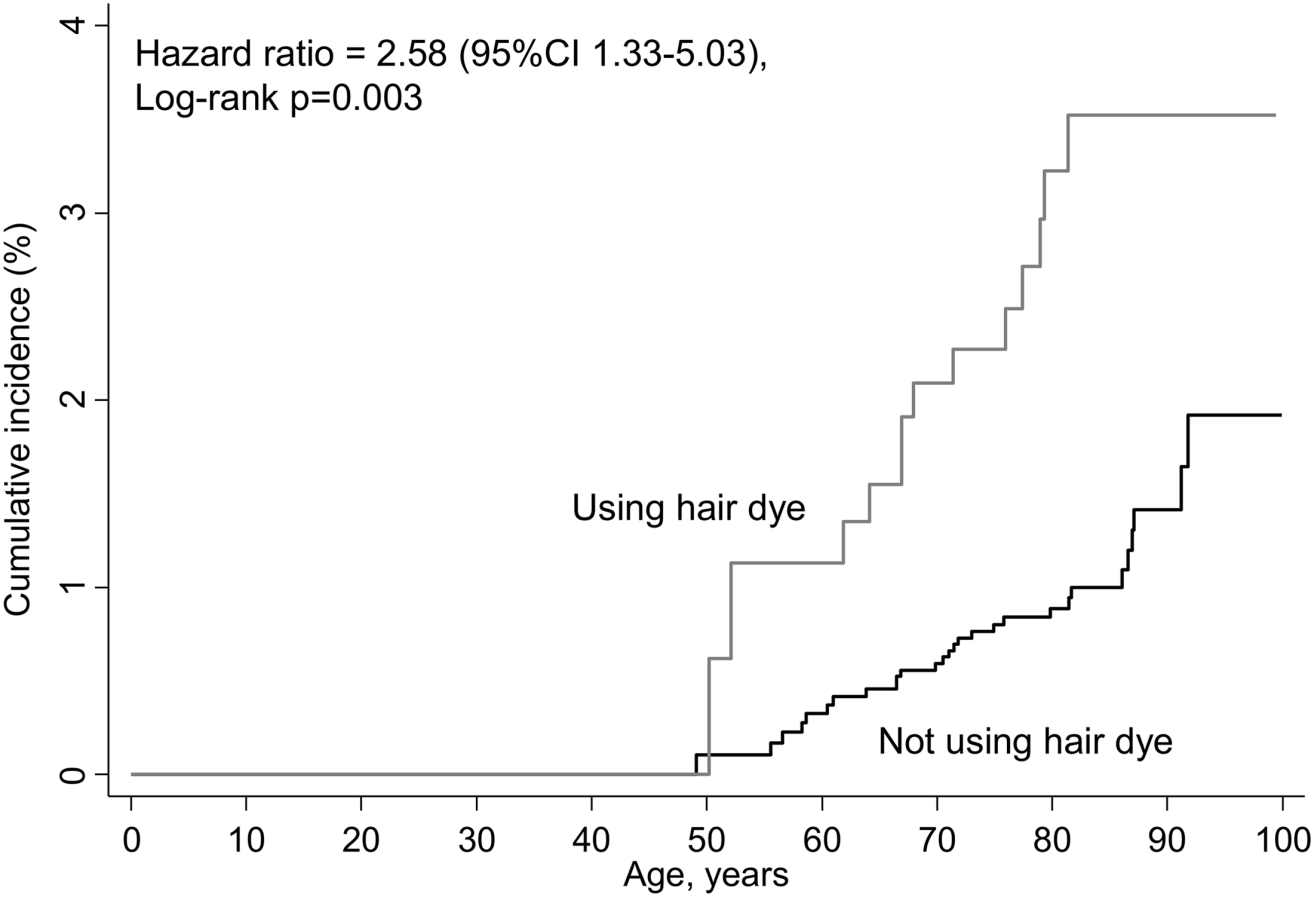
Cumulative incidence of malignant melanoma in women using hair dye compared to women not using hair dye, at baseline as well as at the 1981–1983 examination. In a sensitivity analysis of malignant melanoma, we included participants from the 1981–83 examination who had also attended the 1976–78 examination and had information on the use of personal hair dye from both examinations (n = 5,095). We grouped participants as using hair dye at both examinations (n = 733) and as not using hair dye at both examinations (n = 4,362). Follow-up in this sensitivity analysis began at the 1981–83 examination and ended at event (n = 41), death (n = 3,385), emigration (n = 22) or 1^st^ of January 2012. Age is used as timescale.

We further explored the diagnosis of malignant melanoma in a sensitivity analysis including only the participants who attended both the first 1976–78 and the second 1981–83 examination in the Copenhagen City Heart Study, and who either used hair dye or not at both examinations: the cumulative incidences thus separated even more then when only information on hair dye for the 1976–78 examination was considered (compare Fig 2 with Fig 1). The multivariable adjusted hazard ratio for malignant melanoma in women with versus without personal hair dye use at both examinations was of 2.58 (CI 1.33–5.03), log-rank p-value 0.003.

When looking at the localization of the malignant melanoma, there were no differences between users of hair dye and those who did not use hair dye (See S3 Table).

### Other major morbidities

During follow-up, 93% of all participants received a diagnosis from hospital discharge or ambulatory care of other major morbidities. There was no difference between the two groups regarding all diagnoses (data not shown). We had 80% power to find a minimal detectable hazard ratio of 1.08 over 37 years. We found no convincing difference between the two groups regarding other major morbidities (Table 3). However, we found a slightly increased risk of digestive diseases, with a multivariable adjusted hazard ratio for hair dye users of 1.11 (1.02–1.21), but this was not significant after Bonferroni correction for multiple comparisons.

**Table 3:** Other major morbidities and all-cause mortality according to personal hair dye use during up to 37 years of follow-up in women from the general population.

	Not using hair dye, n = 5,961	Using hair dye, n = 1,723	Hazard ratio(95% confidence interval)	P-value^b^
	No. of events	No. of events		
Infectious and parasitic diseases	922	254	0.91 (0.79, 1.05)	0.18
Respiratory infections	1,244	381	1.00 (0.89, 1.12)	0.98
Diabetes Mellitus^a^	563	140	0.85 (0.70, 1.02)	0.09
Neuropsychiatric conditions	1,887	572	0.99 (0.90, 1.08)	0.77
Cardiovascular diseases	3,627	1,050	0.95 (0.88, 1.02)	0.13
Respiratory diseases	1,476	425	0.90 (0.81, 1.00)	0.06
Digestive diseases	2,261	733	1.11 (1.02, 1.21)	0.01^NS^
Skin diseases	831	235	0.91 (0.79, 1.05)	0.21
Musculoskeletal diseases	2,686	843	1.04 (0.96, 1.12)	0.33
Genitourinary diseases	2,316	746	1.08 (1.00, 1.18)	0.06
Autoimmune diseases	238	67	0.90 (0.68, 1.18)	0.44
Miscarriage	52	11	1.41 (0.72, 2.77)	0.32
All-cause mortality, no. of deaths	4,237	1,227	1.00 (0.94, 1.07)	0.99

Hazard ratios were multivariable adjusted for age, birth year, body mass index, smoking, pack-years, alcohol consumption, leisure-time physical activity, marital status, level of education, level of income, systolic blood pressure, plasma cholesterol, plasma triglycerides, plasma glucose, forced expiratory volume in one second (FEV_1_) % of predicted value, and FEV_1_/FVC.

^a^ Diagnosis of diabetes mellitus was multivariable adjusted, but not for baseline plasma glucose.

^NS^ = Not significant when controlled for 13 multiple comparisons using the Bonferroni method (required p-value = 0.05/13 = 0.004).

^b^ = Wald’s test.

### Mortality

During 192.957 person-years of follow-up, 5,464 women died (71%). There was no difference in all-cause mortality between the two groups. After multivariable adjustments, the hazard ratio for all-cause mortality in women with versus without personal hair dye use was 1.00 (95% confidence interval 0.94–1.07) (Table 3). With a two-sided significance level of 5%, we had 80% power to find a minimal detectable hazard ratio of 1.09 over 37 years.

### Morbidity sub-analyses

In sub-analyses, we did not find any differences in the risk of ischemic heart disease or ischemic cerebrovascular disease between the two groups (Table 4). There were likewise no differences at baseline between the two groups regarding chronic obstructive pulmonary disease or asthma. We further explored the diagnoses of digestive diseases and found a slightly increased risk of gallbladder and biliary tract diseases when using personal hair dye; however, this did not remain significant after Bonferroni correction. Women using hair dye had no excess risk of liver diseases or primary biliary cirrhosis. We did not find any differences regarding diagnoses of atopy, contact dermatitis, or urticaria. Also, in sub-analyses of the women with or without personal hair dye use at both the first 1976–78 and the second 1981–83 examination in the Copenhagen City Heart Study, we found no increase in the reporting of hypersensitivity with the use of personal hair dye. Lastly, we explored two common neuropsychiatric conditions and found an increased risk of Parkinson’s disease among the users of personal hair dye, with a multivariable adjusted hazard ratio of 1.60 (1.08–2.35). Again, this result was not significant after Bonferroni correction. There was no difference in the risk of Alzheimer’s dementia between the two groups.

**Table 4:** Morbidity leading to hospitalization, spirometrically chronic obstructive pulmonary disease, self-reported asthma, and hypersensitivity according to hair dye use during up to 37 years of follow-up.

	Not using hair dye, n = 5,961	Using hair dye, n = 1,723	Hazard/Odds ratio (95% confidence interval)	P-value^c^
	No. of events	No. of events		
**Cardiovascular diseases**				
Ischemic heart disease	1,645	430	0.91 (0.82, 1.01)	0.08
Ischemic cerebrovascular disease	850	253	1.03 (0.89, 1.18)	0.73
**Respiratory diseases**				
Spirometrically defined chronic obstructive pulmonary disease^a^	413	130	1.14 (0.90, 1.45)	0.27
Self-reported asthma^a^	127	36	1.01 (0.68, 1.50)	0.94
**Digestive diseases**				
Liver diseases	160	47	0.97 (0.70, 1.35)	0.87
Primary biliary cirrhosis	33	13	1.29 (0.67, 2.48)	0.44
Diseases of gallbladder and biliary tract	332	123	1.27 (1.03, 1.56)	0.03^NS^
**Skin diseases and allergy**				
Atopy	5	2	1.72 (0.32, 9.31)	0.53
Contact dermatitis	44	6	0.43 (0.18, 1.00)	0.05
Urticaria	44	9	0.70 (0.34, 1.45)	0.34
Self-reported hypersensitivity^b^	1,161	329	1.00 (0.86, 1.15)	0.97
**Neurological and psychiatric conditions**				
Parkinson’s disease	81	38	1.60 (1.08, 2.35)	0.02 ^NS^
Alzheimer’s dementia	171	59	1.11 (0.82, 1.50)	0.49

Hospitalizations according to hair dye use. Relative risks are hazard ratios unless marked with ^a^ or ^b^. All analyses are multivariable adjusted for age, birth year, body mass index, smoking, pack-years, alcohol consumption, leisure-time physical activity, marital status, level of education, level of income, systolic blood pressure, plasma cholesterol, plasma triglycerides, plasma glucose, Forced expiratory volume in one second (FEV_1_) % of predicted value, and ratio of FEV_1_/forced vital capacity.

^a^ Marked analyses are odds ratios calculated at baseline. Analysis of chronic obstructive pulmonary disease at baseline is not adjusted for the ratio of FEV1/FVC.

^b^ Marked analysis is odds ratio of hypersensitivity measured in 1981–83.

^c^ = Wald’s test.

^NS^ = Not significant when controlled for 13 multiple comparisons using the Bonferroni method (required p-value = 0.05/13 = 0.004).

## Discussion

In this study of 7,684 women from the general population with a maximum follow-up of 37 years, we found no major differences in morbidity and mortality among women using personal hair dye and women not using hair dye. Our finding of a 2-fold risk of malignant melanoma in women using hair dye is hypothesis generating. To our knowledge, a study investigating the risk of cancer, skin diseases, other major morbidities, and mortality in the general population with >30 years of follow-up has not been done before.

Mechanistically, our finding of a 2-fold risk of malignant melanoma in women using hair dye likely has a straight forward explanation. The most frequently used hair dyes are the oxidative hair dyes, containing primary intermediates and couplers, which in the presence of hydrogen peroxide infuse color into the hair shaft [5]. Oxidative, also called permanent hair dye contains aromatic amines that are mutagenic in vitro [8], and carcinogenic in animals [33] and humans [1]. Thus, as hair dye often will be in contact with the skin, an increased risk of malignant melanoma seems easy to envision. Interestingly, the latest report from the International Agency for Research on Cancer (IARC) concluded that personal use of hair dye could not be classified as carcinogenic in humans. Since then, two meta-analyses have reported increased risk of bladder cancer [34] and of other types of cancer among hairdressers [35], while a meta-analysis on the personal use of hair dye concluded that there was no excess risk of bladder cancer. However, the risk of other cancer types and the personal use of hair dye are still debated, especially the risk of hematopoietic cancers [36,37]. In our study, we did not find an increased risk of any types of cancer except a 2-fold increase in the risk of malignant melanoma. Although our result for malignant melanoma was not significant after Bonferroni correction for multiple comparisons, we chose to further explore the risk of malignant melanoma in a sub-analysis of women reporting use of personal hair dye at both the first 1976–78 and the second 1981–83 examination of the Copenhagen City Heart Study. Here, the increased risk remained and was in fact even more pronounced, making it difficult to believe that the finding of a 2-fold increased risk of malignant melanoma was merely a chance finding or solely due to confounding.

The association between personal use of hair dye and skin cancer has been assessed before. A case-control study of 511 patients diagnosed with either preinvasive or invasive malignant melanoma and 511 controls found an increased risk of Hutchinson’s melanotic freckle melanoma among users of personal hair dye [38], while a Danish case-control study of 474 cutaneous malignant melanoma patients and 926 controls found no excess risk of malignant melanoma when using personal hair dye [39]. The study found the most important risk factors to be raised naevi, freckling and light hair color as well as exposure to intense sun light which is in accordance other studies [40]. In hairdressers, a large cohort study of 38,866 female hairdressers investigating cancer risk reported a standardized incidence ratio of 2.43 of in situ cancer of the skin at the neck and scalp, which are contact sites for hair dye [41]. Finally, one case-control study of 160 patients diagnosed with basal carcinoma and 200 controls found that these patients tended to use hair dye more frequently, and also to use more dark colors than those without basal cell carcinoma [42]. Taken together with the present findings, it is not possible to exclude an increased risk of malignant melanoma among women using hair dye.

Limitations of the present study need to be considered. The present finding of a 2-fold risk of malignant melanoma in women with versus without personal use of hair dye might be confounded by differences in sun exposure behaviour that we cannot account for. However, we did not find an excess of other skin cancers which are also associated with sun exposure [43], arguing against sun exposure as a confounder in our study. Also, there is some evidence that malignant melanomas of the head and neck are associated with chronic sun exposure, whereas truncal melanomas are associated with intermittent sun exposure and multiple naevi [44]. However, in the present study we did not find any differences in localization of the malignant melanoma according to the use of hair dye. Lastly, we cannot account for differences in skin pigmentation which is another risk factor for developing malignant melanoma [40]. Taken together, although we cannot account for confounders such as sun exposure, naevi, and skin pigmentation, we cannot exclude that women using personal hair dye have an increased risk of malignant melanoma. The increased risk needs confirmation in other studies, preferably with more statistical power than the present study.

We found a small increase in the risk of digestive diseases among the women using hair dye, although this was also not significant after Bonferroni correction. When exploring our finding we found an increased risk of gallbladder and biliary tract diseases, but not of liver diseases or of primary biliary cirrhosis, which have been reported before among users of personal hair dye [45,46]. Mechanistically, we have no explanation for our finding and the result will also need confirmation in other studies.

Interestingly, we found no increase in skin diseases other than malignant melanoma when stratifying by major morbidity causes and no increase specifically in atopy, contact dermatitis, or urticaria in sub-analyses. However, we only have information on skin diseases leading to hospital contacts, and it has previously been shown that despite severe skin reactions after use of hair dye, affected individuals only contact health services in few cases [47]. Therefore, the cases represented in our analyses might not include all cases within our population. We did however in a sub-analysis address the question of hypersensitivity, and found no difference between women using personal hair dye and women not using hair dye.

Lastly, we found an increased risk of Parkinson’s disease among users of personal hair dye, although the result was also not significant after Bonferroni correction. While we have found no studies investigating the use of hair dye and the development of Parkinson’s disease, a number of epidemiological studies have reported an increased risk of malignant melanoma among Parkinson’s disease patients, while others have reported an increased risk of Parkinson’s disease among patients diagnosed with malignant melanoma [48]. The association between the personal use of hair dye and Parkinson’s disease likewise needs confirmation in other studies.

Strengths of the present study include the large sample size and the length of follow-up time. We were able to include 7,684 women from the general population and follow them for a median of 27 years through use of the unique Danish registers. Likewise, we were able to include several covariates measured at baseline influencing mortality and morbidity. Finally, since we used the Danish registers no participants were lost to follow-up.

The study has several limitations. We were not able to divide the participants into ever versus never users of hair dye, as this question was not included in our questionnaire. Secondly, we have no information on type of hair coloring product, meaning oxidative versus non-oxidative, frequency of use, duration of exposure, or color or shade, which could be seen as a proxy for dye load, since darker colors have higher content of aromatic amines [1]. We also do not have any information on sun exposure, naevi, skin pigmentation or hair color, information which could influence our results regarding both malignant melanoma and Parkinson’s disease, since the risk of Parkinson’s disease increases with decreasing darkness of hair [49].

The women participating in the Copenhagen City Heart Study’s first examination in 1976–1978 were most likely exposed to some of the aromatic amines that were discontinued in the 1970’s due to their carcinogenicity in rodents [1]. Given that the most hazardous components of hair dye are now substituted with less hazardous, our findings that the use of hair dye in 1976–1978 does not have major influences on morbidity and mortality is reassuring.

## Conclusion

Personal use of hair dye in women does not have major influences on morbidity and mortality. We found no increased risk of any cancer type except malignant melanoma, although this result was not significant after Bonferroni correction for multiple comparisons. However, in sensitivity analysis of the risk of malignant melanoma, the increase in risk remained. The finding of a 2-fold increased risk of malignant melanoma in women using hair dye is hypothesis generating and should be addressed in detail in other studies.

## Supporting Information

S1 Table Diagnoses leading to hospitalization. Diagnoses are from the national Danish Patient Registry which uses the World Health Organization’s International Classification of Diseases 8^th^ edition from 1978–1993 and 10^th^ edition from 1994 and onwards.
(DOCX)

S2 Table Cancer diagnoses. Diagnoses of cancer are from the Danish Cancer Registry which uses World Health Organization’s International Classification of Diseases 7th and 10th edition.
(DOCX)

S3 Table Localization of malignant melanoma according to personal hair dye use. Data are n (%). P-value is calculated using Fischer’s exact test. Head and neck: C430, C431, C432, C433, C434. Body and extremities: C435, C436, C437, C438.
(DOCX)
