# Supplementary material for: Morbidity and Mortality in 7,684 Women According to Personal Hair Dye Use: The Copenhagen City Heart Study followed for 37 Years
Source: PLoS One. 2016 Mar 17;11(3):e0151636. doi: 10.1371/journal.pone.0151636 (PMC4795553; doi:10.1371/journal.pone.0151636)
Supplement: S1 Table — Diagnoses are from the national Danish Patient Registry which uses the World Health Organization’s International Classification of Diseases 8th edition from 1978–1993 and 10th edition from 1994 and onwards. (DOCX) [file pone.0151636.s001.docx]

|  | **Diagnostic codes** | |
| --- | --- | --- |
|  | **ICD 8^th^ edition** | **ICD 10^th^ edition** |
|  |  |  |
| Infectious and parasitic diseases | 000-136, 320, 323, 612-614, 620, 622 | A00-B99, G00, G03-G04, N70-N73 |
| Respiratory infections | 460-486, 381-382 | J00-J06, J10-J18, J20-J22, H65-H66 |
| Diabetes mellitus | 249, 250 | E10-E14 |
| Neuropsychiatric conditions | 290-315, 321-322, 324-358 | F01-F99, G06-G98 |
| Cardiovascular diseases | 390-458 | I00-I99 |
| Respiratory diseases | 490-519 | J30-J98 |
| Digestive diseases | 530-577 | K20-K92 |
| Skin diseases | 680-709 | L00-L98 |
| Musculoskeletal diseases | 710-738 | M00-M99 |
| Genitourinary diseases | 580-599, 610-611, 621, 623-629 | N00-N30, N60-N64, N75-N98 |
| Autoimmune diseases | 712.1, 712.2, 712.3, 734.1, 695.49, 734.9, 734.00- 734.09, 716 | M05, M06, M32, L93, M35.0, L94, M34, M33 |
| Miscarriage | 643 | O03 |
|  |  |  |
| Ischemic heart disease | 410-414 | I20-I25 |
| Ischemic cerebrovascular disease | 432-435 | I63-I64, G45 |
|  |  |  |
| Liver diseases | 570-573 | K70-K76 |
| Primary biliary cirrhosis | 572 | K74.5 |
| Diseases of gallbladder and biliary tract | 574-576 | K80-K83 |
|  |  |  |
| Atopy | 691 | L20 |
| Contact dermatitis | 692 | L23 |
| Urticaria | 708 | L50 |
|  |  |  |
| Parkinson’s disease | 342.99 | G20, F023 |
| Alzheimer’s dementia | 290.1 | F00.0-F00.9, G30.0-G30.9 |
