# Supplementary material for: Morbidity and Mortality in 7,684 Women According to Personal Hair Dye Use: The Copenhagen City Heart Study followed for 37 Years
Source: PLoS One. 2016 Mar 17;11(3):e0151636. doi: 10.1371/journal.pone.0151636 (PMC4795553; doi:10.1371/journal.pone.0151636)
Supplement: S2 Table — Diagnoses of cancer are from the Danish Cancer Registry which uses World Health Organization’s International Classification of Diseases 7th and 10th edition. (DOCX) [file pone.0151636.s002.docx]

|  | **Diagnostic codes** | |
| --- | --- | --- |
|  | **ICD 7^th^ edition** | **ICD 10^th^ edition** |
|  |  |  |
| Malignant melanoma | 190 | C43 |
|  |  |  |
| Other skin cancers | 191 | C44, D04, D03 |
|  |  |  |
| **Respiratory cancers** |  |  |
| Lung cancer | 162, 164 | C33, C34, C38, D021, D022 |
| Pharynx cancer | 140-149 | C00-11, C13, C14, D000 |
| Larynx cancer | 160, 161 | C12,C30-32, D020 |
| **Gastrointestinal cancers** |  |  |
| Colon cancer | 153, 154 | C18-C21, D010-D013 |
| Pancreas cancer | 157 | C25 |
| Liver cancer | 155, 156 | C22-C24, D015 |
| Stomach cancer | 151 | C16, D002 |
| Oesophagus cancer | 150 | C15, D001 |
| Small intestine cancer | 152 | C17 |
| **Hematological cancers** |  |  |
| Leukemia | 204,207 | C91-C95 |
| Multiple myeloma | 203, 205 | C88, C90 |
| Non-Hodgkin’s lymphoma | 200, 202 | C82-C85, C96 |
| Hodgkin’s lymphoma | 201 | C81 |
| **Women’s cancers** |  |  |
| Breast cancer | 170 | C50, D05 |
| Ovary cancer | 175, 176 | C51, C52, C56, C57, D070-D073 |
| Corpus uteri cancer | 172-174 | C54, C55 |
| Cervix cancer | 171 | C53, D06 |
| **Other cancers** |  |  |
| Urinary cancers | 181 | C65-C68, D090 |
| Kidney cancer | 180 | C64 |
| Brain cancer | 192, 193 | C69-C72 |
| Sarcoma | 196, 197 | C40, C41, C45-C49 |
| Endocrine cancers | 194, 195 | C37, C73-C75, D093 |
| Remaining cancers | 158, 159, 179, 199 | C26, C39, C58, C60, C63, C76, C80, D014, D017, D019, D023, D024, D07, D09 |
