# Supplementary material for: Morbidity and Mortality in 7,684 Women According to Personal Hair Dye Use: The Copenhagen City Heart Study followed for 37 Years
Source: PLoS One. 2016 Mar 17;11(3):e0151636. doi: 10.1371/journal.pone.0151636 (PMC4795553; doi:10.1371/journal.pone.0151636)
Supplement: S3 Table — Data are n (%). P-value is calculated using Fischer’s exact test. Head and neck: C430, C431, C432, C433, C434. Body and extremities: C435, C436, C437, C438. (DOCX) [file pone.0151636.s003.docx]

|  | **No. of participants not using hair dye**  **n = 40** | **No. of participants using hair dye**  **n = 25** | **P-value** |
| --- | --- | --- | --- |
|  |  |  |  |
| Head and neck | 5 (12.5%) | 2 (8%) | 0.70 |
| Body and extremities | 35 (87.5%) | 23 (92% ) |  |
|  |  |  |  |
